# Knowledge and practices of preconception care among rural Japanese women: findings from a mixed methods investigation

**DOI:** 10.1186/s12884-023-05940-8

**Published:** 2023-09-16

**Authors:** Yasumi Shibata, Michiko Abe, Keiichiro Narumoto, Makoto Kaneko, Nobuko Tanahashi, Michael D. Fetters, Machiko Inoue

**Affiliations:** 1 Morimachi Family Medicine Clinic, Morimachi, Shizuoka Japan; 2 Hamamatsu University School of Medicine Hospital General Medical Training Program Shizuoka. Family Medicine Training Program, Hamamatsu, Japan; 3 London Iryo Center, London, UK; 4 Department of Family and Community Medicine, Hamamatsu University School of Medicine, Hamamatsu, Japan; 5 Department of Obstetrics, Gynecology and Family Medicine, Hamamatsu University School of Medicine, Hamamatsu, Japan; 6 Department of Health Data Science, Yokohama City University, Yokohama, Japan; 7 Department of Family Medicine, University of Michigan, Ann Arbor, USA; 8 The School of Health Humanities, Peking University Health Science Center, Beijing, China

**Keywords:** Preconception care, Women’s health, Reproductive health, Family planning, Primary care

## Abstract

**Background:**

Preconception care is not widespread in Japan and there is a pressing need to improve the practice. The present study assessed the knowledge and behavior of preconception care among women to seek effective intervention. Our research questions were: 1) How much do women know about preconception care? 2) How much are they practicing preconception care and what are the information sources of their behavior? 3) Do the women's preconception care behavior associated with accurate knowledge?

**Methods:**

The research was conducted in a rural town in central Japan. Using an exploratory sequential mixed methods design, we undertook interviews, developed a survey based on the qualitative results, and then conducted a survey. The interviews explored how preconception care was perceived and practiced in women of childbearing age. The survey was designed to investigate the knowledge of preconception care among women with and without pregnancy experience, their practice behavior of preconception care, and whether the behavior is associated with knowledge.

**Results:**

The participants were 13 for the interview and 232 for the survey. They had limited access to preconception care recommendations and advice for specific actions was given by obstetricians and gynecologists after pregnancy. There was a large gap in knowledge about preconception care between parous and nulliparous women, especially about the need for folic acid supplementation. Practices that were manageable in their daily lives, such as cessation of smoking and alcohol, diet, and weight management, were considered common sense. In contrast, recommended practices that require medical attention, such as screening for sexually transmitted diseases and cervical cancer, tended to be less accurately known and practiced. Participants' sources of information about preconception care were the Internet, family and friends and mass media.

**Conclusion:**

In rural Japan, women of childbearing age lack knowledge about preconception care, especially before their first pregnancy. Primary care providers should try outreach to schools and women’s groups in the community, promote information sharing among family and close friends, and utilize information technology to enhance the knowledge and practice of preconception care.

## Background

Awareness about the essentiality of preconception care for improving the health of mothers and children is growing globally [1–3]. Preconception care is defined as “the provision of biomedical, behavioral, and social health interventions to women and couples before they become pregnant [4]”. Currently, there are high maternal and perinatal mortality rates due to low nutrition, anemia, smoking, and other health complications among women giving birth, and around 40% of pregnancies are unintended [4]. Therefore, interventions to enhance the health management behavior of women and families must initialize before pregnancy [3]. Specific items suggested as “preconception care” include adequate folic acid intake, cessation of smoking and alcohol, maternal health management such as maintaining proper weight, eating a balanced diet, prevention of sexually transmitted diseases, early detection of human papillomavirus infection (HPV) and rubella infections, screening for cervical cancer, and family planning [4, 5]. Healthcare professionals, on the one hand, have mutual knowledge about the concept of preconception care, but regional variations exist in the aspect of penetration of practicing it. For example, official recommendations for preconception care started in the United States in 2006 [1] and in the UK in 2008 [6]. The promotion of preconception care in low-income countries has been lower, where the health care attention is much on saving lives [3].

Japan has one of the safest perinatal care systems in the world [7, 8]. However, there is a pressing need to promote preconception care. A Preconception Care Center to disseminate the concept in the country was not opened by the National Center for Child Health and Development [9] until 2014. Despite having safe perinatal care, problems exist. The occurrence of neural tube defects was 3.6 cases per 10,000 deliveries in 1990, and it increased to 5.2 in 2012 [10]. The increase may be due to insufficient folic acid intake as only 8% of women consume it before pregnancy [11]. The rate of low birth weight infants is rising, reaching 9.4% in 2018, higher than in any other advanced country [12, 13]. Further concern originates from approximately 13% to 21% of women in their 20s to 40s being underweight [14]. Furthermore, the rate of cervical cancer screening among women in their 20s to 40s remains around 45% [15] considerably lower than in advanced countries [16] even though screening once every two years is recommended and funded by the government [17]. The reported syphilis cases have constantly increased since 2010, particularly for women in their 20s [18]. The number of other sexually transmitted diseases has not been mitigated in the recent past [19]. Furthermore, the Japan Environment and Children’s Study, which is a nationwide cohort study with samples of approximately 100,000 unique mothers, indicated regional differences in the rate of obese and underweight pregnant women and smoking rates in the early stages of pregnancy [20, 21]. These trends require interventions for young women and families to boost their health management behavior for potential future pregnancy. Community-based techniques are needed to respond to the unique characteristics of each region.

Preconception care covers a wide range of items and often involve preventive medical practices and lifestyle modifications. Despite high expectations for primary care providers to implement preconception care, it has not yet become a part of daily primary care practice [22, 23]. According to the Health Belief Model [24], to promote preventive health behavior, people need to be enlightened about the risk of developing a condition or disease, and support in lowering their beliefs about the tangible and psychological costs of practicing them. To devise effective interventions for preconception care from the field of primary care, researchers, clinicians, and policymakers need to understand appropriately women's views toward preconception care. However, most studies have fixated on one item or category of a wide range of preconception care [25, 26]. Furthermore, prior studies have used questionnaires to seek women's knowledge or behavior for preconception care. These quantitative techniques lack information about women's nature, including their needs, expectations, and barriers to accessing services [26–28]. To fix this gap, in this research, we sought to investigate women's views and experiences about preconception care by conducting interviews with women of the target age and then developing a questionnaire based on the interview outcomes. Our research questions were: 1) How much do women know about preconception care? 2) How much are they practicing preconception care and what are the information sources of their behavior? 3) Do the women's preconception care behavior associated with accurate knowledge? We sought to comprehend preconception care not only to expand the medical body of knowledge but also to understand the context of women’s reality and to employ the findings to develop efficient interventions for the community.

## Methods

### Design

**Fig. 1:**
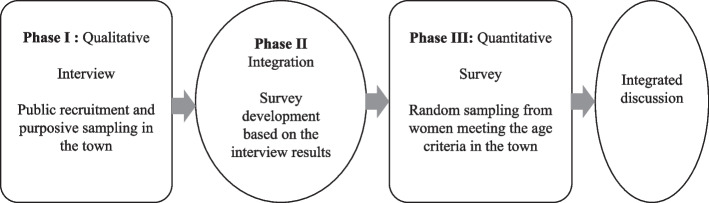
The procedural diagram of the exploratory sequential mixed methods survey development research design

The aim of the present study is to investigate women's views and experiences about preconception care. Based on the paradigm of pragmatism, we employed an exploratory sequential mixed methods survey development research design with three phases [29]. Phase 1 entailed a qualitative strand using semi-structured interviews to understand the participants’ perspectives. Phase 2 comprised questionnaire development based on the Phase 1 interview results. Phase 3 involves deploying the questionnaire from Phase 2 using an online format. The questionnaire was designed to investigate the knowledge of preconception care among women with and without pregnancy experience, their practice behavior of preconception care, and whether the behavior is associated with knowledge. Finally, meta-inferences were discussed as integrated findings of both studies. Figure 1 depicts an overview of the procedural diagram of the research and detailed methods are described in each phase section.

### Human subjects research approval

Participants gave written consent in the interviews and the survey indicating their agreement of the voluntary nature of study participation and the freedom to withdraw from the study. Human subjects review and ethical approval was acquired from The Institutional Review Board of Hamamatsu University School of Medicine in Japan [No. 18–06 and 19–172].

### Setting

We conducted this research in a rural town in central Japan. Logging, tea growing, ceramics and crafts production, and a local hospital serve as the pillars of the local economy. Like many rural locations in Japan, the town has an aging population. Among the population of less than 20,000 and still declining, the pool of individuals for potential participation in this study was about 1800 women of childbearing age ranging from 20 to 43 years old. They had the benefit of having a public family medicine clinic that provides comprehensive primary care including women’s health care. We chose this setting because we anticipated that the physicians in the clinic could regionally address the issues detected in this study by working with local counterparts.

## 【Phase I: Qualitative Interviews】

## Materials and methods

### The data collection instrument

We developed an interview guide to explore how preconception care is perceived and practiced in the aspect of participants' daily lives or their life course [30]. The overarching constructs in the guide included knowledge about preconception care, behavior related to preconception care, and sources of information about preconception care. The questions included frequency of using health care facilities, memorable experiences of pregnancy and childbirth (for women with pregnancy experience only), plans and hopes for future pregnancies, knowledge and practice of preconception care, and expectations for preconception care implemented by health care providers.

### Recruitment and sampling

We placed flyers at the town hall, the public hospital, and the family medicine clinic to recruit participants. We offered each participant a gift certificate equivalent to US$30 as a symbol of our gratitude for their participation and compensation for their time. We intentionally sampled to enroll in roughly equal representation of women who never had pregnancy (nulliparous women) and women who have been pregnant (parous women). Initially, we received no interest from nulliparous women, so we directed clinical staff to recruit participants from their personal networks for this demographic.

### Data collection

We performed interviews from November 2018 to March 2019. The interviewers included family physicians (YS, NT), and a qualitative researcher majoring in intercultural communication (MA), all of whom were women and had no connection to the participants before the interview. The participants’ privacy was guaranteed by using a closed meeting room in the clinic. Interviews took roughly 30–60 min.

### Analysis

For the qualitative data analysis, we wrote a 3Cs summary [31] immediately after each interview, which included context, content, and concepts from the interview. We shared these among co-researchers to conduct an iterative analysis subsequently. Interview recordings were transcribed verbatim, and we performed a thematic analysis [32] by inductive coding. MAXQDA Analytic Pro 12 was used for data organization and management. We conducted interviews with a total of 13 participants until the research team members supported the saturation of findings as the endpoint for data collection. For member checking [33], an individual summary was distributed to each participant with overall study outcomes. We solicited their opinion about their views being represented and any further comments on the content, and informed if they do not respond by the deadline, we would assume it as their approval. Eight of the 13 participants responded in coherence with the study outcomes. No additional comments were received.

## Results–qualitative findings

We interviewed 13 women aged 22–43 years. The mean age among them was 30.9 years, with seven parous women who were married (mean age of 35.4 years), and six nulliparous women who were unmarried (mean age of 25.6 years).

Among preconception care items, we classified those that indicated similar trends in participants’ knowledge and behavior and found five thematic areas: folic acid intake, cessation of smoking and alcohol, weight management and nutritionally balanced diet, rubella vaccine, and screening for cervical cancer and sexually transmitted infections.

### Folic acid intake

There was an evident difference in folic acid knowledge depending on whether the participants previously had a pregnancy. Parous women knew they should consume folic acid and had used the supplement. This action was directly proposed to participants by obstetricians, pharmacists, family members, and friends, as well as through media such as maternity magazines, the Internet, and pamphlets. However, most participants took folic acid supplements during pregnancy. They were unaware that they should have begun taking it before pregnancy and the rationale. Some discontinued the supplements' consumption because they found them cumbersome or thought they could derive folic acid from their daily diet.

Nulliparous women were unfamiliar with the term “folic acid.” Unlike parous women, single women and married women who were not at a stage in their life of undergoing family planning had few opportunities to learn about folic acid.

### Cessation of smoking and alcohol

Regardless of the prior pregnancy experience, most of the participants conformed to “common sense” that smoking and drinking alcohol harm pregnancy. Among parous women, many were not regular smokers or drinkers of alcohol, and they said it was not difficult for them to abstain from these behavior, including during lactation.

Some participants related their experience of miscarriage to their smoking habits before the pregnancy or discussed feeling guilty for not being able to stop smoking during pregnancy. A few participants complained that they had experienced passive smoking, or their colleagues and family members were unsupportive of quitting smoking. Such participants’ thoughts implied that they were aware of the dangers of smoking to the fetus.

### Weight management and a nutritionally balanced diet

Parous women indicated receiving guidance at the hospital during pregnancy on weight and nutrition management. They deliberated strategies for not becoming overweight by exercising and increasing vegetable intake. However, this guidance took place after their pregnancy and was not outlined as preconception care.

Many nulliparous women did not actively think about nutritional management, and some still relied on their parents for meal preparation. One participant had received advice from her family physician to take care of her menstrual disorder and overweight problems. But it was not functioning as preconception care because she was not intending to get pregnant.

We found that participants valued being thin during the interview. The gap between the young women’s esthetics and “appropriate weight” in preconception care was concerned.

### Rubella vaccine

Most parous women were recommended by the hospital to receive rubella antibody testing during their pregnancy and recognized the need for vaccination before the next pregnancy. Cost subsidies from the local government were described by participants as encouraging them for getting rubella vaccination. However, in some cases, the participants forgot to seek vaccination after giving birth or could not find how to receive a cost subsidy.

The problem of rubella infection during pregnancy was recognized even among nulliparous women. When conducting interviews, there was a rubella outbreak in Japan, and the media cautioned about the lack of official vaccination recommendations for men born between 1962 and 1978. Because all the study participants were women, most reported that they had been vaccinated in their childhood, but they were unsure why the vaccination is required before pregnancy.

### Screening for cervical cancer and sexually transmitted infections

All participants, parous and nulliparous, recognized that women over 20 years of age were eligible for periodic cervical cancer screening. Vouchers distributed by the local government, recommendations in the workplace, and public advertisements on television helped to offer this knowledge.

Parous women discussed receiving cervical cancer screening during pregnancy or as one of the tests during fertility treatment. In this aspect, the physician arranges the test items so that women’s attitudes toward the tests were somewhat passive. On the other hand, the advice from a close friend or the presence of a relative who experienced cervical cancer influenced them to take the test regularly by their will.

Most nulliparous women had never been to an obstetrician or gynecologist. They did not know what a pelvic examination encompasses, which seemed to be a major reason for their hesitancy to perform screening tests.

Like cervical cancer screening, parous women recognized sexually transmitted infection screening as part of fertility treatments and pregnancy tests. Nulliparous women knew about sexually transmitted infections from their school education but did not take it as a personal risk. The reason for screening test hesitation was discovered to be the same as cervical cancer screening.

### Summary of qualitative findings

In summary, the qualitative study observed differences in knowledge of preconception care for many items depending on whether or not the participant had pregnancy experience. Even those who had the knowledge and had been practicing some aspects of preconception care did not understand the reasons, and they were greatly unaware that they should initialize such practices before pregnancy. Furthermore, parous women who had an uncomplicated pregnancy and delivery with their first child did not consistently engage in preconception care, as they anticipated doing well with their second and subsequent children.

## 【Phase II: Survey Development: Women’s Health and Pregnancy Survey】

## Materials and methods

### Initial development

We developed the Women’s Health and Pregnancy questionnaire based on the themes and hypotheses collected from the Phase I interviews and prior research [34–39]. The instrument was implemented to evaluate six primary domains: concerns for women (5 items), preconception care practice (10 items), perceptions about pregnancy (21 items), knowledge of preconception care (20 items), and health status (3 items), as well as 11 demographic items. We referred to Primary Care Obstetrics and Gynecology (PCOG) (2017) [5] and Farahi & Zolotor (2013) [40] as proper and reliable guidance for organizing preconception care knowledge and practice behavior.

### Cognitive testing

We conducted cognitive testing [41] with two non-medically trained women chosen to be comparable to the intended participants. They first completed the entire questionnaire, and a team member checked item by item whether they understood each question as intended and modified any wordings that were not interpreted as intended. This process resulted primarily in wording changes to enhance the clarity of items. We reduced the number of knowledge questions from 20 to 14 to balance the number of questions between practice categories (weight management, folic acid supplementation, etc.) and updated some wording to avoid double barreled questions.

### Pilot survey

We undertook a pilot test with knowledge questions for 51 women of reproductive age outside the research area. Three items were revised because of the high correct answer rate (> 90%), which implied low discriminatory ability. These questions were: “No. 3. High blood pressure is not a problem for pregnancy (reverse question) (correct answer rate 90.2%)” was changed to “Even if you have chronic diseases, managing your health before pregnancy can promote a good pregnancy outcome”, “No. 4. There are some medicines that are safe to take during pregnancy, but some medicines are not safe (90.2%)” to “If you take cold medicines during pregnancy, you must have an abortion (reverse question)” and “No. 13. Rubella infection during pregnancy can cause fetal abnormalities (96.1%)” to “Rubella vaccinations can be given at any time during pregnancy (reverse question)”.

The final survey included current life conditions (3 items), perceptions about pregnancy (7 items), preconception care practices (9 items), knowledge of preconception care (14 items), as well as 11 demographic items including five health status items. We administered the survey over the Internet using Google forms. Given that the purpose of the survey was for single use in the current study, formal validity and reliability testing were not performed.

## Results–integration of qualitative results and survey items

**Fig. 2:**
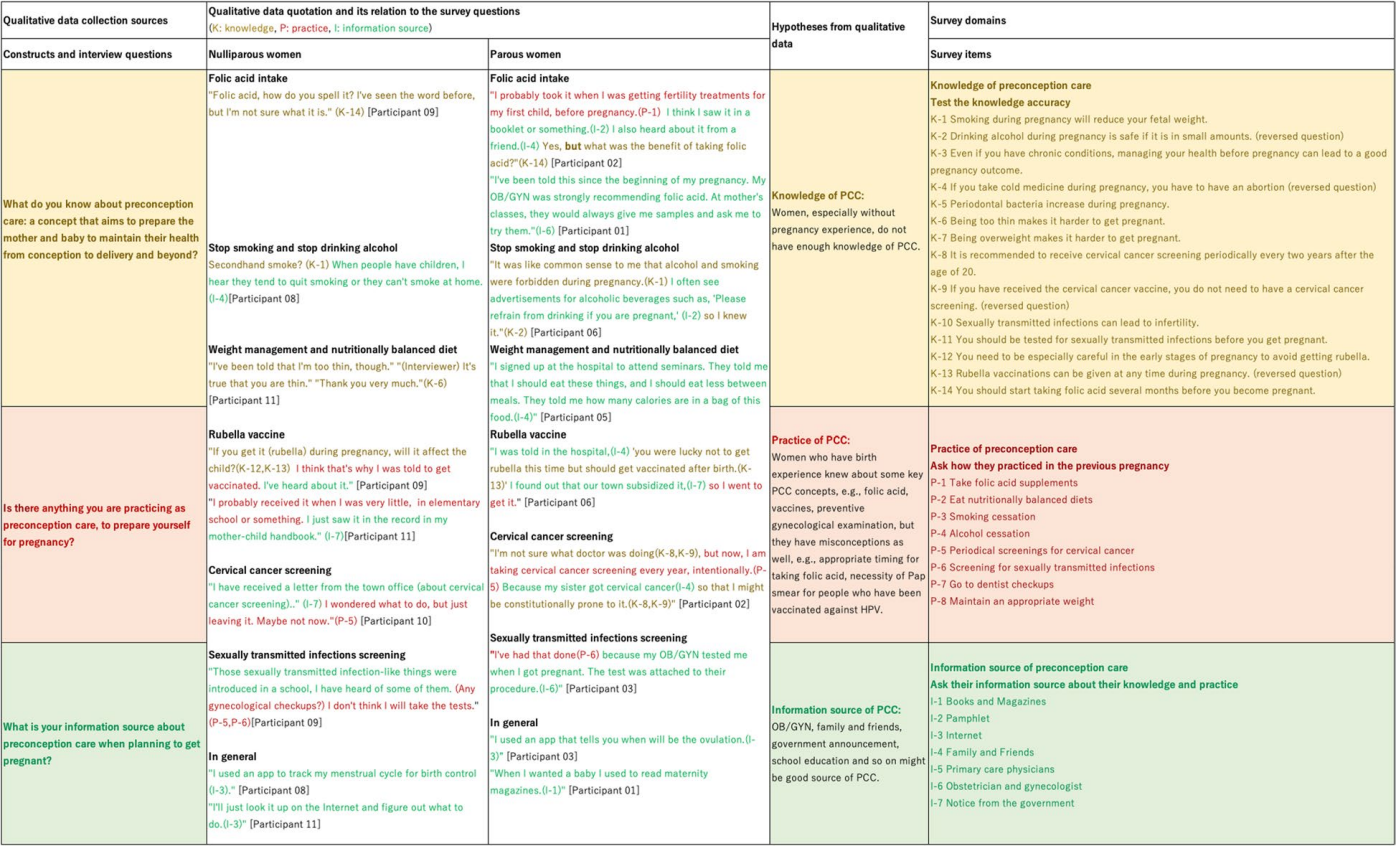
Joint display planning: qualitative findings and survey structure. PCC represents preconception care. Colors of the columns and words indicate themes such as knowledge in yellow, behavior in red, and information sources in green

In the current study, we fixated our analysis on participants’ knowledge and practices about preconception care and how it connects to their pregnancy experience. Figure 2 depicts a joint display of our qualitative findings and the survey items we created [42]. On the left side, our focused themes are listed with colors that are knowledge in yellow, behavior in red, and information sources in green. In the following two columns, major quotes from Phase I interviews by preconception care item were expressed. The columns were classified into parous women and nulliparous women. Since the elements of the three themes were merged in each quote, we categorized the words by theme colors. The middle of the table describes the hypothesis we derived from the qualitative data analysis. The right side of the table indicates the structure of the questionnaire. We developed knowledge tests of preconception care to check the knowledge of the target population, questions to ask about the experience of practicing preconception care for parous women and a question about information sources about preconception care with listed options based on the interview data.

## 【Phase III: Survey】

## Materials and Methods

### Survey administration

From 1662 women meeting the age criteria, the municipal government implemented a list in descending order of ages between 20 and 43 years and provided a list of 831 contacts created from every second woman. We sent an invitation once in April 2020 by postcard with a URL and QR code that was linked to the online survey. The invite implied that the first 200 respondents would receive a gift certificate equivalent to US$5.

### Analysis

We conducted a descriptive analysis for the preconception care knowledge test, practice behavior, and information sources. Then we conducted chi-square tests regarding the associations between the knowledge and pregnancy experience, and the associations between the knowledge and practice behavior for parous participants, to assess the hypotheses obtained from a qualitative study.

### Mixed data analysis

The interview outcomes were also employed in making meta-inferences about the survey. We contrasted these survey outcomes with the hypotheses derived from the first Phase interview and then drew meta-inferences.

## Results – survey findings

### Participants' characteristics

In total, 232 women responded to the survey (response rate of 26%). Table 1 depicts the survey participants' characteristics. Their median age was 33 (IQR 28–37) years. Parous women numbered 140 (60%), 5 (2%) were pregnant when responding to the survey, and 87 (38%) were nulliparous women. Approximately 60% had been married and had a child/children. Thirteen percent of the women reported experiencing infertility treatment. Regarding knowledge about preconception care, 79% of participants indicated that they did not know the details or did not know about it at all. Among nulliparous women, 98% were unfamiliar with “preconception care.”

**Table 1:** Participants' characteristics

Total number of participants		232	(Response rate 26%)
Median age (IQR)		33 (28–37)	
Education	Junior high school	8	(3%)
	High school	67	(29%)
	College/vocational school	91	(39%)
	University or above	66	(28%)
Occupation status	Full time	117	(50%)
	Part-time	61	(26%)
	Housewife	38	(16%)
	Student	6	(3%)
	None	5	(2%)
	Others	9	(4%)
Marriage status	Married	137	(59%)
	Unmarried	84	(36%)
	Divorced or widowed	11	(5%)
Pregnancy experience	Have experience (Parous)	140	(60%)
	Currently pregnant	5	(2%)
	No experience (Nulliparous)	87	(38%)
Number of children	0	93	(40%)
	1	40	(17%)
	2	58	(25%)
	3	35	(15%)
	4 or more	6	(3%)
Fertility treatment experience	Have experience	30	(13%)
	Currently under treatment	1	(0%)
	No experience	197	(85%)
	No response	4	(2%)
Familiarity with preconception care	Know the content very well	3	(1%)
	Know some of the contents	49	(21%)
	Do not know the details	95	(41%)
	Do not know it at all	87	(38%)

### Research Question 1: How much do women know about preconception care?

Table 2 expresses the women’s knowledge about preconception care, which contrasts parous and nulliparous women. Overall, the percentage of those who had correct answers to the knowledge test was higher among parous women. The items that properly answered rate were significantly higher (*p* < 0.001) among parous women were, in descending order: Q12. You need to be particularly careful in the early stages of pregnancy to avoid getting rubella (93% vs. 67%), Q4. If you take cold medicine during pregnancy, you have to have an abortion (reversed question) (91% vs. 64%), Q1. Smoking during pregnancy will reduce your fetal weight (72% vs. 45%), and Q5. Periodontal bacteria increase during pregnancy (62% vs. 26%). Among nulliparous women, the correctly answered rate for several items was significantly low, in ascending order: Q14. You should start taking folic acid several months before you become pregnant (22%), Q5. Periodontal bacteria increase during pregnancy (26%), and Q1. Smoking during pregnancy will lower your fetal weight (45%).

**Table 2:** Correct answers for preconception care knowledge tests (*n* = 232)

	Total		Pregnancy experience				*P* value
			Parous		Nulliparous		
	*n* = 232 (%)		*n* = 145 (%)		*n* = 87 (%)		
The average number of correct answers on a 14-point scale (SD)^a^	9.09	(3.12)	9.79	(2.60)	7.97	(3.56)	< 0.001
(1) Smoking during pregnancy will reduce your fetal weight	143	(62)	104	(72)	39	(45)	< 0.001
(2) Drinking alcohol during pregnancy is safe if it is in small amounts. (Reversed question)	175	(75)	110	(76)	65	(75)	0.844
(3) Even if you have chronic conditions, managing your health before pregnancy can promote a good pregnancy outcome	137	(59)	88	(61)	49	(56)	0.512
(4) If you take cold medicine during pregnancy, you have to have an abortion (Reversed question)	188	(81)	132	(91)	56	(64)	< 0.001
(5) Periodontal bacteria increase during pregnancy	113	(49)	90	(62)	23	(26)	< 0.001
(6) Being too thin makes it harder to get pregnant	119	(51)	70	(48)	49	(56)	0.090
(7) Being overweight makes it harder to get pregnant	130	(56)	84	(58)	46	(53)	0.452
(8) It is recommended to receive cervical cancer screening periodically every two years after the age of 20	127	(55)	80	(55)	47	(54)	0.865
(9) If you have received the cervical cancer vaccine, you do not need to have a cervical cancer screening. (Reversed question)	177	(76)	114	(79)	63	(72)	0.282
(10) Sexually transmitted infections can lead to infertility	172	(74)	107	(74)	65	(75)	0.877
(11) You should be tested for sexually transmitted infections before you get pregnant	189	(81)	122	(84)	67	(77)	0.176
(12) You need to be particularly careful in the early stages of pregnancy to avoid getting rubella	193	(83)	135	(93)	58	(67)	< 0.001
(13) Rubella vaccinations can be administered at any time during pregnancy. (Reversed question)	131	(57)	88	(61)	43	(50)	0.133
(14) You should start taking folic acid several months before you become pregnant	115	(50)	96	(66)	19	(22)	< 0.001

^a^t-test, others are chi-square tests

Items properly answered by less than 60% for all participants in ascending orders were: Q5. Periodontal bacteria increase during pregnancy (overall 49%), Q14. You should start taking folic acid several months before you become pregnant (overall 50%), Q6. Being too thin makes it harder to get pregnant (overall 51%), Q8. It is recommended to receive cervical cancer screening periodically every two years after the age of 20 (overall 55%), Q7. Being overweight makes it harder to get pregnant (overall 56%), Q13. Rubella vaccinations can be administered at any time during pregnancy (reversed question) (overall 57%), and Q3. Even if you have chronic conditions, managing your health before pregnancy can promote a good pregnancy outcome (overall 59%).

### Research Question 2: How much are they practicing preconception care and what are the information sources for their behavior?

**Fig. 3:**
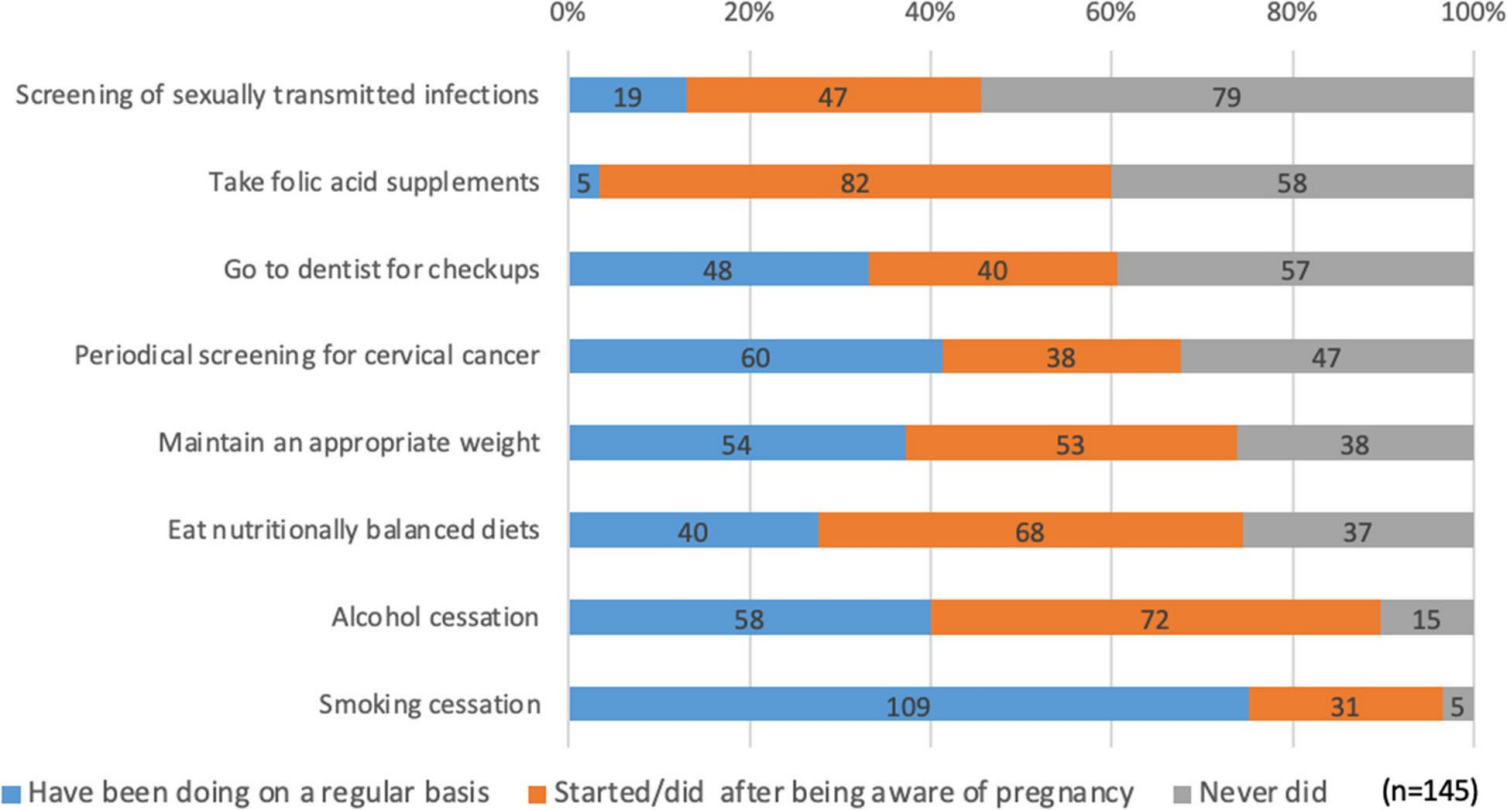
The experience of preconception care behavior among parous women (*n* = 145)

Figure 3 highlights reports of practicing preconception care among the 145 parous women. The least practiced preconception behavior in ascending order were: screening for sexually transmitted infections (46%), taking folic acid intake (60%), having dental checkups (61%), and completing cervical cancer screening (68%). When focusing on the breakdown of "Have been doing on a regular basis" and "started/did after being aware of pregnancy" for each behavior, folic acid intake was rarely performed before pregnancy (3%), while cervical cancer screening and dental screening were performed to some extent (41%, 33%).

More than 90% of the respondents reported a cessation of smoking and alcohol after becoming aware of the pregnancy. About 75% of the respondents reported they practiced consuming a nutritionally balanced diet and maintaining an appropriate weight. Smoking cessation was commonly practiced since the pre-pregnancy period (75%), while alcohol cessation, nutrition, and weight management were only practiced after pregnancy.

**Fig. 4:**
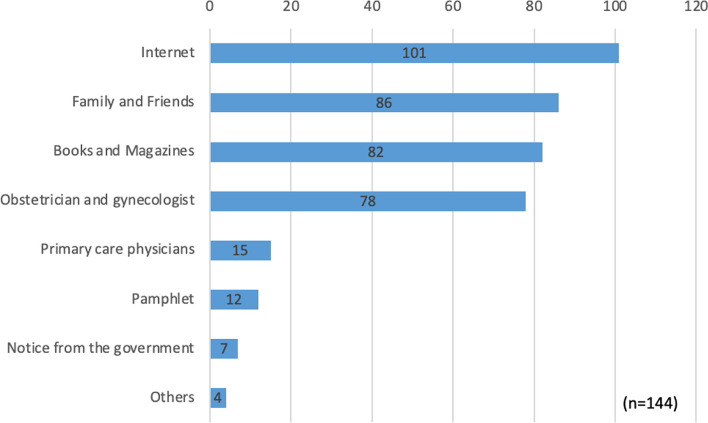
Information sources about preconception care among parous women (*n* = 145)

Figure 4 illustrates the information sources that helped them with their prior pregnancy. Most of the participants mentioned the Internet as an information source (70%), followed by family and friends (60%), books and magazines (57%), and obstetricians and gynecologists (54%). The presence of primary care physicians was low (10%) compared with obstetricians and gynecologists.

### Research Question 3: Do the women's preconception care behavior associated with accurate knowledge?

Table 3 indicates the correlation between knowledge and practices among parous women. There was a correlation between knowledge and practice in “folic acid intake,” “periodical cervical cancer screenings,” and “screening of sexually transmitted infections.” The items that did not conform to knowledge and practice were smoking and alcohol cessation, eating nutritionally balanced diets and maintaining a proper weight, and dental care.

**Table 3:** Correlations between knowledge and practice of preconception care among parous women (*n* = 145)

	Preconception care items	Practiced in the previous pregnancy n (%)		Knowledge test items	Results				*p-*value
					Correct n (%)		Incorrect/ Not sure n (%)		
Lifestyle management	Smoking cessation	140	(97)	(1) Smoking during pregnancy will reduce your fetal weight	100	(96)	40	(98)	0.676
	Alcohol cessation	130	(90)	(2) Drinking alcohol during pregnancy is safe if it is in small amounts (Reversed question)	99	(90)	31	(89)	0.809
	Eat nutritionally balanced diets	108	(74)	(6) Being too thin makes it harder to get pregnant	52	(74)	56	(75)	0.958
	Eat nutritionally balanced diets	108	(74)	(7) Being overweight makes it harder to get pregnant	62	(74)	46	(75)	0.827
Weight management	Maintain an appropriate weight	107	(74)	(6) Being too thin makes it harder to get pregnant	52	(74)	55	(73)	0.896
	Maintain an appropriate weight	107	(74)	(7) Being overweight makes it harder to get pregnant	60	(71)	47	(77)	0.447
Folic acid intake	Take folic acid supplements	87	(60)	(14) You should start taking folic acid several months before you become pregnant	73	(76)	14	(29)	< 0.001
Cervical cancer	Periodical screening for cervical cancer	98	(68)	(8) It is recommended to receive cervical cancer screening periodically every two years after the age of 20	60	(75)	38	(58)	0.034
	Periodical screening for cervical cancer	98	(68)	(9) If you have received the cervical cancer vaccine, you do not need to have a cervical cancer screening (Reversed question)	82	(72)	16	(52)	0.032
Sexually transmitted infections	Screening of sexually transmitted infections	66	(46)	(10) Sexually transmitted infections can lead to infertility	55	(51)	11	(29)	0.017
	Screening of sexually transmitted infections	66	(46)	(11) You should be tested for sexually transmitted infections before you get pregnant	60	(49)	6	(26)	0.041
Dental care	Go to dentist for checkups	88	(61)	(5) Periodontal bacteria increase during pregnancy	55	(61)	33	(60)	0.894

### Summary of overall results

The majority of women were unfamiliar with the concept of preconception care. We found large gaps in knowledge between nulliparous and parous women. The gap was most obvious for folic acid, which they rarely started taking before pregnancy. Among parous women, behaviors that require medical attention were not well practiced, such as screening for sexually transmitted infections, dental checkups, and cervical cancer screening. Some behaviors were well practiced, such as cessation of smoking and alcohol, nutrition, and weight management. However, whether practiced or not, they did not adequately understand the reasons for the best practices, and opportunities to obtain such information are limited. Women's sources of information for preconception care were the Internet, family and friends, and mass media, surpassing the influence of obstetricians/gynecologists and primary care physicians.

## Discussion

The current study investigated preconception care knowledge and behavior among women of childbearing age in a rural area of Japan. We detected great knowledge gaps between nulliparous and parous women. Previous studies of women's knowledge and behavior regarding preconception care have investigated the specific items (e.g., folic acid, smoking, alcohol) using questionnaires [11, 26, 37–39, 43, 44]. In Japan, most studies have focused on medical students [36, 37, 43] and pregnant women [43, 44]. In comparison, the current study used random sampling for women living in the study district and included comprehensive preconception care items. In addition, interviews were conducted in the first phase to understand how preconception care fits into in women's lives from their perspective [27]. These approaches revealed that there were differences in the accuracy of knowledge and level of practice for each of the preconception care items and whether they had experience of pregnancy or not. To encourage women to engage in preconception care behaviors, interventions should be delivered at an appropriate time in an individual's life stage [45, 46], considering the unique barriers for each recommended practice. Effective methods for communicating this knowledge to women and families are discussed below.

First, promoting information sharing among women can have a positive impact on knowledge acquisition and effective implementation [39, 43]. In this study, the knowledge of folic acid intake among nulliparous women was low, and the rate of taking folic acid supplements before pregnancy was only 3.5%. The result regarding the knowledge was comparable to that of Mikamo et al. (2012), where the participants included nulliparous nursing students and the rate of women who knew the need for folic acid intake before pregnancy was around 20% [37]. In terms of practice behavior, our outcome was much worse than that of overall Japan, where the rate was 8% [11]. However, our qualitative study revealed that advice from mothers and close female friends was beneficial when initiating folic acid supplementation. According to Nagusa & Sasaki (2020), women's behavior regarding folic acid intake improved after a three-month preconception care educational intervention that included a seminar, group discussion, and some follow-up interaction [47]. Such educational opportunities which include learning with peers in addition to advice from health professionals, may influence information sharing among women and lead to their behavior change.

Second, healthcare professionals may need to communicate with the community to share the idea of preconception care. This is especially important for younger generations, to reduce barriers to accessing clinical services. Our study observed two types of barriers for women in practicing cervical cancer and sexually transmitted infection screening. One is a lack of knowledge, as these behavior and knowledge were related, and the other is the hesitance of young women to undergo pelvic examinations. Kanamasa et al. (2018) described that even medical students in Japan tend to be hesitant to see a gynecologist due to embarrassment and anxiety [48]. There is a need to inform young people about the risks of infectious diseases, why they should be screened for preconception care, how gynecological examinations are conducted [49], and that there are public services to lower their financial burden. Primary care providers should be able to provide such interventions, but another concern in Japan is that more than 50% of the young generation aged 18–39 do not have a primary care physician [50]. Therefore, primary care providers need to extend opportunities to communicate this issue to broader targets outside the clinic. As an example in the research area, Ito et al. (2014) undertook an intervention study in which family physicians educated middle school students about HPV vaccination and cervical cancer screening, and improved the knowledge of both young girls and their mothers [51]. Partnering with school-based education or women’s community can be a technique to reach younger generations and spread knowledge about preconception care to their friends and family.

Third, we need to recognize the impact of mass media on women's perceptions of their health and lifestyle management. Intriguingly, the well-practiced behavior in the current study such as smoking cessation and alcohol cessation were not correlated with accurate knowledge, but women received messages from the media that such behavior were detrimental to pregnancy. When the media disseminate medically appropriate information, it can be highly effective. However, the opposite can also happen. For example, we were concerned about participants' preference for thinness in the interview, and the correct answer rate for the weight management questions in the survey knowledge test was low, approximately 50%. Prior studies have highlighted that Japanese female students are exposed to media pressure to lose weight from middle school to high school levels [52] and that the negative effects on pregnancy are not understood by them [37]. Thus, when media-reinforced values conflict with preferred interventions for women's health [52, 53], healthcare providers need to evaluate women's perceptions and update their knowledge about what is required for a healthy pregnancy.

Finally, to promote women's access to preconception care, we should focus more on the presence of the women's health industry. In this study, participants who practiced preconception care made the most use of the Internet as a source of information. In recent years, Female Technology, also known as the “femtech” industry, has been growing extensively around the world and comprises services, products, and software that support the health management and well-being of women at different stages of life [54]. A Japanese menstrual cycle management app called ”Lunaluna” has reached 17 million downloads since its inception in 2000 [55]. Barker (2018) highlights the need for social movements and political will to support interventions and engagement with market interests to improve preconception nutrition management in women [45]. The same can be said for preconception care in general. Emerging femtech platforms are a compatible tool for delivering preconception care information to women who frequently do not come to the clinic until they become pregnant. Healthcare providers may need to collaborate with these companies that are developing technologies to offer right knowledge about women’s health, family planning, and preconception care.

This study had some limitations. First, the response rate was low. This may be because of the sensitive nature of the subject matter, and participants may be biased toward those who are more inclined to the topic. Second, survey respondents may tend to over-report socially desirable behavior, and under-report undesirable behavior. However, the strength of this study is that the target participants were randomly chosen from the residents of the research area, which enabled us to survey a wide range of ages and women with and without pregnancy experience. Furthermore, we conducted a comprehensive study of preconception care and detected the areas in which the knowledge and practice behavior correlate. This study is highly original in its focus on Japan, and the rural setting. Critical suggestions were made concerning the current knowledge and behavior of preconception care among the target population and the techniques that can be taken to reach them.

## Conclusion

In rural Japan, women of childbearing age lack knowledge about preconception care. The incentive to be exposed to preconception care recommendations is low, particularly among women before their first pregnancy. The behavior that should be reinforced for the target population are starting folic acid intake before the pregnancy, updating the idea of proper weight for a healthy pregnancy, and the importance of screening for cervical cancer and sexually transmitted diseases. Recommendations from close friends and family members could encourage women to engage in preconception care. Primary care providers should try outreach to schools and women’s groups in the community, and work with modern technology to enhance the knowledge and practice of preconception care.

## Data Availability

The data presented in this study are available from the corresponding author upon reasonable request. The data are not publicly available to protect participants’ privacy.

## Abbreviations

HPV: Human papillomavirus
PCOG: Primary Care Obstetrics and Gynecology
PCC: Preconception care
